# Fabrication and osteogenic differentiation performance of the electrospun magnetic P(VDF-TrFE)/Fe_3_O_4_ composite fibrous membranes

**DOI:** 10.1039/d6ra01554a

**Published:** 2026-05-27

**Authors:** Na Qiang, Lihong Huang, Wenlong Zhang, Rouping Zheng, Yan Yu, Jiao Zou, Qingyun Tang, Yubo Zou, Jinyu Yang, Guocong Liu, Shuai Qiu, Shuo Tang

**Affiliations:** a School of Chemistry and Materials Engineering, Huizhou University Huizhou 516007 China gcl_109@hzu.edu.cn yangjinyu@hzu.edu.cn; b Department of Orthopaedics, The Eighth Affiliated Hospital, Sun Yat-sen University Shenzhen 517000 China tangshuo1205@163.com qiush23@mail.sysu.edu.cn

## Abstract

The design of magnetoactive hybrid scaffolds based on biocompatible piezoelectric polymers and magnetic nanoparticles (NPs) holds great promise for advanced bone tissue regeneration applications. In this study, electrospun magnetoactive poly(vinylidene fluoride-co-trifluoroethylene) [P(VDF-TrFE)] composite fibrous membranes doped with different concentrations of oleic acid-modified Fe_3_O_4_ (Fe_3_O_4_-OA) NPs were successfully fabricated *via* the electrospinning technology. The as-prepared P(VDF-TrFE)/Fe_3_O_4_-OA composite membranes exhibited a uniform fibrous morphology with an average fiber diameter of approximately 0.2 µm, and the piezoelectric crystalline β-phase was predominant. The homogeneous dispersion of Fe_3_O_4_-OA NPs endowed the composite fibrous membranes with typical ferromagnetic behaviour and tunable saturation magnetisation. *In vitro* biological evaluations confirmed the excellent biocompatibility of the composite membranes with bone marrow mesenchymal stem cells (BMSCs). CCK-8 assays indicated that the viability of BMSCs cultured on all composite membrane groups increased over time, and live/dead staining further verified that all composite membranes supported high BMSC viability. F-actin staining results revealed that BMSCs could effectively attach and spread on the composite membranes with well-developed cytoskeletal structures. Moreover, the osteogenic differentiation potential of the BMSCs on the composite membranes was systematically evaluated at the early, middle and late stages of osteogenic induction. Alkaline phosphatase (ALP) activity assays revealed that the composite membranes containing Fe_3_O_4_-OA NPs exhibited significantly elevated ALP activity compared with the pure P(VDF-TrFE) membrane, and RUNX2 immunofluorescence staining confirmed enhanced early osteogenic transcriptional activation in the BMSCs cultured on the Fe_3_O_4_-OA-doped composite membranes. Collectively, these findings suggest that the electrospun magnetic P(VDF-TrFE)/Fe_3_O_4_-OA composite fibrous membranes exhibit good cytocompatibility and support the osteogenic differentiation of BMSCs, indicating their potential as scaffold materials for bone tissue engineering.

## Abstract 2

Fe_3_O_4_-OA was incorporated into P(VDF-TrFE) fibers *via* electrospinning. The resulting membranes exhibited a bone-like topological structure and physiological surface potential, showing cytocompatibility and great potential for bone defect repair.

## Graphical abstract

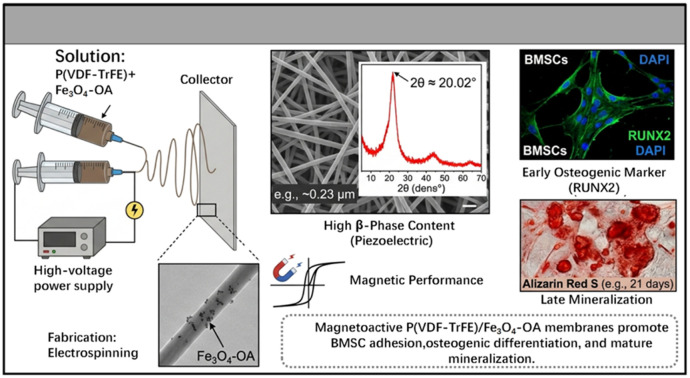

## 1. Introduction

Electrical signals, as the medium for nerve impulses, enable the real-time transmission of biological information. Consequently, the application of human–computer interaction interfaces for the transmission of external electrical signals and bioelectrical signals has attracted considerable attention.^1,2^ Exploring appropriate technical means and methods to establish effective electrical transmission and interaction interfaces is a research hotspot in materials science and neuroscience. In the field of neural tissue regeneration, external electrical stimulation is also regarded as a powerful tool for effectively promoting recovery from nerve injury. The development of effective techniques for applying electrical stimulation to nerve cells and tissues has broad application prospects for regulating neural functions, recording neural activities and promoting neural regeneration.^3,4^ However, traditional electrical stimulation is conducted *via* implanted electrodes. Their invasiveness and continuous contact with neural tissues are prone to cause interface effects, secondary damage and inflammatory responses. Thus, an urgent need exists to develop effective non-invasive radio stimulation techniques.

Piezoelectric materials can generate electrical energy *via* mechanical conversion and are most likely to enable biosafe electrical stimulation applications without requiring implanted electrodes or external power sources.^5–7^ However, the piezoelectric effect requires mechanical stimulation or ultrasonic action, limiting its application in patients or areas with movement disorders. In addition, its application is restricted because the piezoelectric effect induced by ultrasonic stimulation can have adverse effects on tissues due to poor tissue penetration of ultrasonic waves and the potential for cavitation and thermal effects. Magnetoelectric materials composed of piezoelectric and magnetically responsive materials can generate electrical stimulation in response to a mild external magnetic field, effectively avoiding the problems stated above.^8,9^ These materials have attracted increasing attention from researchers. A typical magnetoelectric stimulation platform can achieve electrical output under magnetic stimulation by adding magnetic particles to piezoelectric polymer films.

Among the piezoelectric material systems that have been explored, single-component and multi-component inorganic crystal compounds occupy an important position because of their excellent properties. Typical representatives include lithium niobate (LiNbO_3_),^10,11^ zirconate titanate (PZT)^12,13^ and barium titanate (BaTiO_3_).^14,15^ Although these inorganic materials exhibit excellent piezoelectric properties, their inherent drawbacks cannot be ignored, such as PZT posing potential environmental and health risks. Further, they display insufficient mechanical toughness and are prone to brittle fracture. Meanwhile, the material preparation process requires complex techniques, such as high-temperature sintering, which increases energy consumption and costs.

In contrast, biocompatible piezoelectric polymers are highly promising alternative materials because of their unique advantages. The material combines high flexibility and lightweight properties and can be fabricated into various functional devices *via* gentle processes, such as solution processing and hot pressing. Among numerous biocompatible piezoelectric polymers, polyvinylidene fluoride (PVDF) and its copolymers stand out particularly.^16,17^ Notably, PVDF needs to be induced to form an electroactive β crystal phase through specific treatment, while its copolymer with trifluoroethylene, poly(vinylidene fluoride-co-trifluoroethylene) [P(VDF-TrFE)], has a naturally stable β crystal phase structure resulting from the steric hindrance effect of the trifluoroethylene units. This structural stability renders P(VDF-TrFE) an ideal matrix material for constructing magnetoelectric composite systems.^18,19^ When constructing magnetic active composites, magnetite (ferriferrous oxide, Fe_3_O_4_) nanoparticles (NPs) with high saturation magnetization have become the preferred magnetic fillers because of their excellent magnetic response characteristics.^20,21^ The combination of the two provides a new direction for developing multifunctional intelligent materials.

In the field of regenerative medicine, synthetic scaffolds are the core materials for tissue engineering applications because of their customisable physicochemical properties. Electrospinning is a traditional and efficient preparation method that can precisely control the microstructure and mechanical properties of the scaffold by regulating process parameters.^22–24^ Notably, when constructing a composite scaffold with both piezoelectric and magnetic properties, the mechanical stress and electric field effects generated during the electrospinning process can effectively promote the formation and orientation of the β crystal phase of the piezoelectric polymer, significantly enhancing the material's electrical activity.^25,26^ Previous research showed that oleic acids (OA) modify Fe_3_O_4_ NPs to achieve a uniform distribution of magnetic fillers.^27,28^ This property stems from the stabilising effect of the carboxyl group in OA, which inhibits NPs agglomeration *via* charge repulsion and enhances the compatibility of the system.

Recent advancements have highlighted the potential of P(VDF-TrFE)/Fe_3_O_4_ magnetoelectric scaffolds as non-invasive platforms for tissue engineering. While prior studies have demonstrated the individual benefits of piezoelectricity and magnetism, a critical gap remains in identifying the precise filler loading that synergistically optimizes these properties without compromising biological efficacy. In this study, we hypothesize that the strategic incorporation of oleic acid-modified Fe_3_O_4_ (Fe_3_O_4_-OA) nanoparticles can simultaneously modulate the polymeric crystalline structure and provide a tunable magnetoelectric microenvironment. We specifically address this by systematically investigating the correlation between Fe_3_O_4_-OA concentration and the resulting β-phase content, *d*_33_ coefficient, and saturation magnetization. This research provides new insights into the “structure–property–biofunction” relationship, demonstrating how molecular-level interfacial interactions between modified nanoparticles and the P(VDF-TrFE) matrix govern the robust osteogenic differentiation of BMSCs, from initial cytoskeletal remodeling to late-stage mineralization. This study is expected to provide a theoretical and experimental basis for tissue engineering.

## 2. Materials and methods

### 2.1 Materials

P(VDF-TrFE) was purchased from Shanghai Aichun Biotechnology Co., Ltd (Shanghai, China). Fe_3_O_4_ (20 nm) was purchased from Aladdin (Shanghai, China). The other reagents used in this experiment were purchased from Huizhou Nanyuan Chemical Products Co., Ltd (Huizhou, China).

### 2.2 Characterization

#### 2.2.1 Scanning electron microscopy (SEM)

The samples were sputter-coated with gold and visualized *via* scanning electron microscopy (SEM) (JSM-6380LA Analytical SEM, JEOL Ltd, Tokyo, Japan), operated at an accelerating voltage of 5 kV. The nanofiber diameters were calculated as the mean of 100 filaments of each sample, measured from different SEM images using the ImageJ software (1.44p) of the National Institutes of Health (NIH) (Bethesda, MD, USA).

#### 2.2.2 Fourier transform infrared (FTIR) analysis

The treated and untreated samples were ground, and FTIR spectra were recorded using a KBr pellet in an infrared spectrometer (TENSOR27, German).

#### 2.2.3 X-ray diffraction (XRD) analysis

The crystal structures of the samples were investigated using an X-ray powder diffractometer (Ultima IV, Rigaku, Tokyo, Japan) with Cu Kα radiation (*λ* = 1.5406 Å and 40 kV, 40 mA).

#### 2.2.4 Thermo gravimetric analysis (TGA)

The thermal decomposition temperature was investigated using a thermogravimetric analyzer (TG 209 F1, Netzsch, Selb, Germany) purged with nitrogen. The analysis was carried out over a temperature range from 35 °C to 800 °C at a scanning rate of 10 °C min^−1^ and a N_2_ flow of 20 mL min^−1^.

#### 2.2.5 Differential scanning calorimetry (DSC)

The crystallization behavior of the polymers was investigated using a modulated differential scanning calorimeter (MDSC 2910, TA Instruments, New Castle, DE, USA) purged with nitrogen. DSC was carried out over a temperature range from 0 °C to 200 °C at a scanning rate of 10 °C min^−1^.

#### 2.2.6 *d*_33_

Piezoelectric coefficient was measured using a Berlincourt-type quasistatic *d*_33_ meter (model ZJ-3A; IACAS, Beijing, China).

#### 2.2.7 Vibration sample magnetization (VSM)

The *M*–*H* curves for the P(VDF-TrFE)/Fe_3_O_4_-OA composite electrospun membranes were evaluated by carrying out VSM (JDAW-2000D, Changchun Yingpu Magnetoelectric Technology Development Co., LT, Changchun, China) at room temperature.

#### 2.2.8 Cell culture

Human bone marrow-derived mesenchymal stem cells (BMSCs) were cultured in DMEM (C11885500BT, Gibco) supplemented with 10% fetal bovine serum (FBS) (10099141C, Gibco) and 1% penicillin–streptomycin (15140122, Gibco) under standard conditions (37 °C, 5% CO_2_). The culture medium was refreshed every two days. Cells at passages 3–5 were used for all experiments to ensure phenotypic consistency.

Prior to cell culture, the electrospun membranes were sterilized by immersion in 75% ethanol for 24 h, followed by thorough rinsing with phosphate-buffered saline (PBS) to remove residual ethanol. The samples were then pre-incubated in a complete culture medium for 24 h to improve cell compatibility and subsequently placed into culture plates for cell seeding. A static magnetic field of approximately 150 mT was applied during the cell culture experiments. Cells cultured on the composite membranes were continuously maintained under these magnetic field conditions throughout the incubation period. All other experimental conditions were kept consistent.

#### 2.2.9 Cell proliferation assay

The cytocompatibility of the composite membranes was evaluated using a Cell Counting Kit-8 (CCK-8) assay (Dojindo, Shanghai, China). BMSCs were seeded onto membranes with different Fe_3_O_4_-OA contents at a density of 1 × 10^4^ cells per well. After 1, 3, and 7 days of incubation, the culture medium was replaced with fresh medium containing 10% CCK-8 reagent and incubated for an additional 2 h. The absorbance was measured at 450 nm using a microplate reader. All experiments were performed in triplicate.

#### 2.2.10 Live/dead staining

Cell viability on the composite membranes was further assessed by live/dead staining. After 3 days of culture, cells were washed with phosphate-buffered saline (PBS), incubated with calcein-AM/propidium iodide (C2015S, Beyotime, China) for 15 min, washed again, and imaged using a fluorescence microscope (Axio Observer 7, Zeiss, Germany).

#### 2.2.11 Cytoskeletal organization and cell spreading analysis

BMSCs cultured on the membranes for 3 days were rinsed with PBS and fixed with 4% paraformaldehyde for 10 min at room temperature. Then, the samples were permeabilized with 0.1% Triton X-100 for 5 min and washed thrice with PBS. The F-actin was stained using phalloidin-Alexa Fluor 555 (C2203S, Beyotime, China), and cell nuclei were detected using DAPI (G1012, Servicebio, China). All these images were visualized with a fluorescence microscope (Axio Observer 7, Zeiss, Germany).

#### 2.2.12 Alkaline phosphatase (ALP) activity assay

BMSCs cultured on the membranes were subjected to osteogenic induction after initial attachment. The culture medium was replaced with osteogenic induction medium, which was refreshed every 2 days. After osteogenic induction for 3 and 7 days, the cells were washed in phosphate-buffered saline (PBS) three times and then lysed using a cell lysis buffer for Western blotting and IP without inhibitors (P0013J, Beyotime, China), and the lysates were measured using a commercial ALP assay kit (P0321, Beyotime) following the manufacturer's protocol. The absorbance was measured at 405 nm and normalized to the total protein content.

#### 2.2.13 Immunofluorescence staining of RUNX2

For immunofluorescence analysis, BMSCs cultured on the membranes with osteogenic induction for 3 and 7 days were washed in PBS and fixed with 4% paraformaldehyde for 10 min at room temperature. The samples were then permeabilized with 0.1% Triton X-100 for 10 min and washed thrice with PBS. Then, the samples were blocked with 3% (w/v) bovine serum albumin and incubated with the primary antibody, RUNX2 Polyclonal antibody (20700-1-AP, Proteintech, China), overnight at 4 °C, followed by incubation with a fluorescently labeled secondary antibody. Cell nuclei were stained with DAPI. All these images were visualized with confocal microscopy (LSM980, Zeiss, Germany).

#### 2.2.14 Western blot analysis

Total proteins were extracted from BMSCs cultured on the membranes following osteogenic induction, and RUNX2 and OPN were evaluated at 7 and 14 days. Equal amounts of protein were separated by SDS–PAGE and transferred onto PVDF membranes. After blocking, membranes were incubated with primary antibodies against RUNX2 (20700-1-AP, Proteintech, China) or OPN (30200-1-AP, Proteintech, China), followed by HRP-conjugated secondary antibodies (RGAR001, Proteintech, China). Protein bands were visualized using enhanced chemiluminescence, and GAPDH (GB15004, Servicebio, China) was used as the internal control.

#### 2.2.15 Alizarin Red S staining

Alizarin Red S staining was performed on days 14 and 21. Cells cultured on the membranes were fixed with 4% paraformaldehyde for 10 min and subsequently stained using an Alizarin Red S Staining Kit for Osteogenesis (C0148S, Beyotime, China) for 1 h. After staining, the cells were washed three times with distilled water prior to observation.

### 2.3 Methods

#### 2.3.1 Preparation of the P(VDF-TrFE) electrospinning solution

P(VDF-TrFE) powder was dissolved in *N*,*N*-dimethylformamide (DMF) and acetone (3 : 2). P(VDF-TrFE) accounted for 10% of the total solution mass fraction. The solution was heated and stirred in a water bath at 40 °C until it was completely dissolved.

#### 2.3.2 Fe_3_O_4_ modified with OA

Fe_3_O_4_ (0.5 g) was added to 100 mL of deionised water and subjected to ultrasonication for 20 minutes. The magnetic suspension was poured into a round-bottomed flask and mechanically stirred for 10 minutes. Then, 1 mL of OA and 5 mL of ethanol were added to the round-bottomed flask. The solution was heated to 75 °C and stirred for 3 hours. The solution was decanted, and an appropriate amount of anhydrous ethanol was added, causing Fe_3_O_4_ to settle. A magnet was used to collect Fe_3_O_4_. Finally, it was dried in an oven at 75 °C for 10 hours.

#### 2.3.3 Preparation of the P(VDF-TrFE)/Fe_3_O_4_ electrospinning solution

A set mass of Fe_3_O_4_ NPs was weighed and added to the fully dissolved P(VDF-TrFE) electrospinning solution. Ultrasonic oscillation was performed in an ice-water bath for 1 hour to ensure uniform dispersion. Electrospinning solutions containing unmodified Fe_3_O_4_ were prepared at mass concentrations of 0%, 4%, 8% and 12%. In addition, electrospinning solutions with modified Fe_3_O_4_ mass concentrations of 4%, 8% and 12% were prepared.

#### 2.3.4 Electrospinning

The prepared electrospinning solution was collected and transferred to a syringe. Various P(VDF-TrFE) composite membranes with different contents of Fe_3_O_4_-OA were spun using the ET-2535X electrospinning equipment. The electrospinning parameters were adjusted as follows: the electrospinning voltage was 12 kV, the liquid supply rate was 1 mL h^−1^, and the receiving distance was 10 cm. Membrane thickness was regulated by controlling the electrospinning time. The spinning time was set at 15 minutes in this experiment.

### 2.4 Statistical analysis

Data are presented as mean ± standard deviation (SD) from three to six independent experiments. Analyses were performed with GraphPad Prism v9. One-way ANOVA and two-way ANOVA with Tukey's post hoc test were used for group comparisons. Significant differences were defined as *p < 0.05, **p < 0.01, ***p < 0.001 and ****p < 0.0001; ns means no significance.

## 3. Results and discussion

### 3.1 Effect of different Fe_3_O_4_ concentrations on the morphology of electrospun membranes

**Fig. 1:**
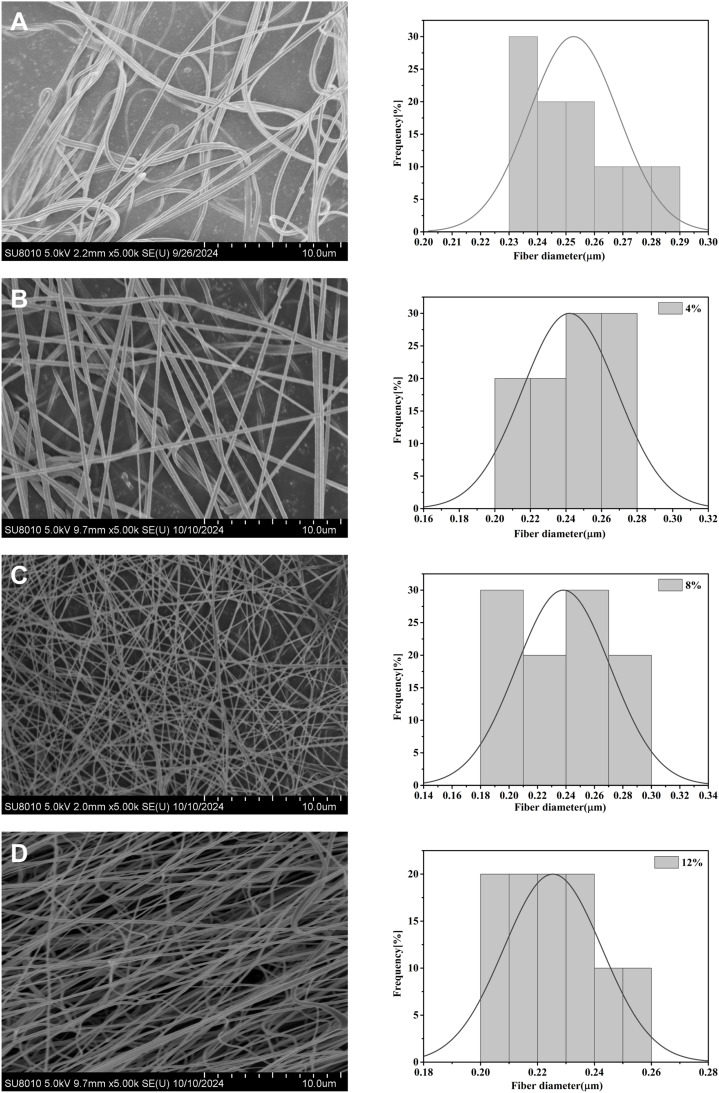
SEM images and fiber diameter distributions of P(VDF-TrFE) with unmodified Fe_3_O_4_ at concentrations of (A) 0%, (B) 4%, (C) 8%, and (D) 12%.

When the mass concentration of Fe_3_O_4_ was increased from 4%, 8% and further to 12%, the average diameter of the electrospun fibers decreased from 0.25 µm and 0.24 µm to 0.22 µm, respectively (Fig. 1). These results indicated a clear tendency for the fiber diameter to decrease with an increase in Fe_3_O_4_ concentration. After incorporating Fe_3_O_4_ NPs, the conductivity of the electrospinning solution was enhanced by the inherent electrical conductivity of Fe_3_O_4_. During the electrospinning process, the increased conductivity led to a strong electric field force acting on the spinning solution jet, in turn resulting in great nanofiber stretching, yielding a reduced fiber diameter.^29^ In terms of fiber arrangement, all the as-prepared nanofibers exhibited a disordered and interlaced structure. In addition, the surface of the P(VDF-TrFE)/Fe_3_O_4_ composite nanofibers was relatively rough, whereas the surface of the pure P(VDF-TrFE) nanofibers was smooth. This difference in surface morphology might be attributable to the rigid nature of Fe_3_O_4_ as an inorganic NP. When Fe_3_O_4_ NPs were mixed into the organic P(VDF-TrFE) solution, the surface roughness of the resulting composite nanofibers was significantly enhanced. Collectively, these morphological characteristics confirmed that Fe_3_O_4_ NPs were successfully incorporated into the P(VDF-TrFE) nanofiber matrix.

**Fig. 2:**
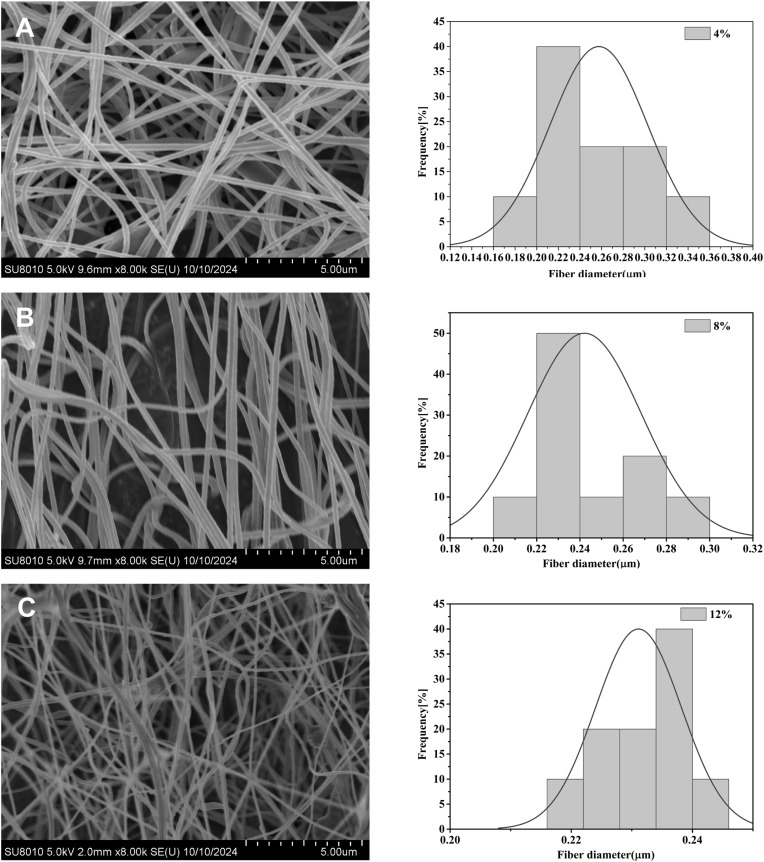
SEM images and fiber diameter distributions of P(VDF-TrFE) with modified Fe_3_O_4_ at concentrations of (A) 4%, (B) 8%, and (C) 12%.

Due to the high surface energy of the Fe_3_O_4_ nanoparticles (NPs), they tend to aggregate to reduce their surface energy.^30^ Such aggregation is unfavourable for the uniform dispersion of Fe_3_O_4_ NPs in the electrospinning solution, thereby impairing the overall performance of the composite material. To address this agglomeration issue, Fe_3_O_4_ NPs were modified with OA. Fig. 2 shows that when the mass concentration of Fe_3_O_4_ was fixed at 4%, 8%, or 12%, the average diameter of the composite nanofibers prepared with OA-modified Fe_3_O_4_ NPs was larger than that of the nanofibers without Fe_3_O_4_ modification, with values of 0.25 µm, 0.24 µm and 0.23 µm, respectively. This phenomenon was attributable to the surface properties of the modified Fe_3_O_4_ NPs: the surface of Fe_3_O_4_ NPs modified by OA was coated with hydrophobic alkyl long chains. When these modified NPs were dispersed in the electrospinning solution, the alkyl long chains entangled with the P(VDF-TrFE) polymer molecules to a certain extent, leading to an increase in the viscosity of the electrospinning solution. During the electrospinning process, the initial viscosity of the polymer jet directly influenced the stretching effect induced by the electric field; a high initial viscosity resulted in the high resistance of the jet to stretching. Consequently, the stretching degree of the polymer jet was reduced, ultimately leading to an increase in the diameter of the electrospun nanofibers.

### 3.2 X-ray diffraction (XRD) analysis

**Fig. 3:**
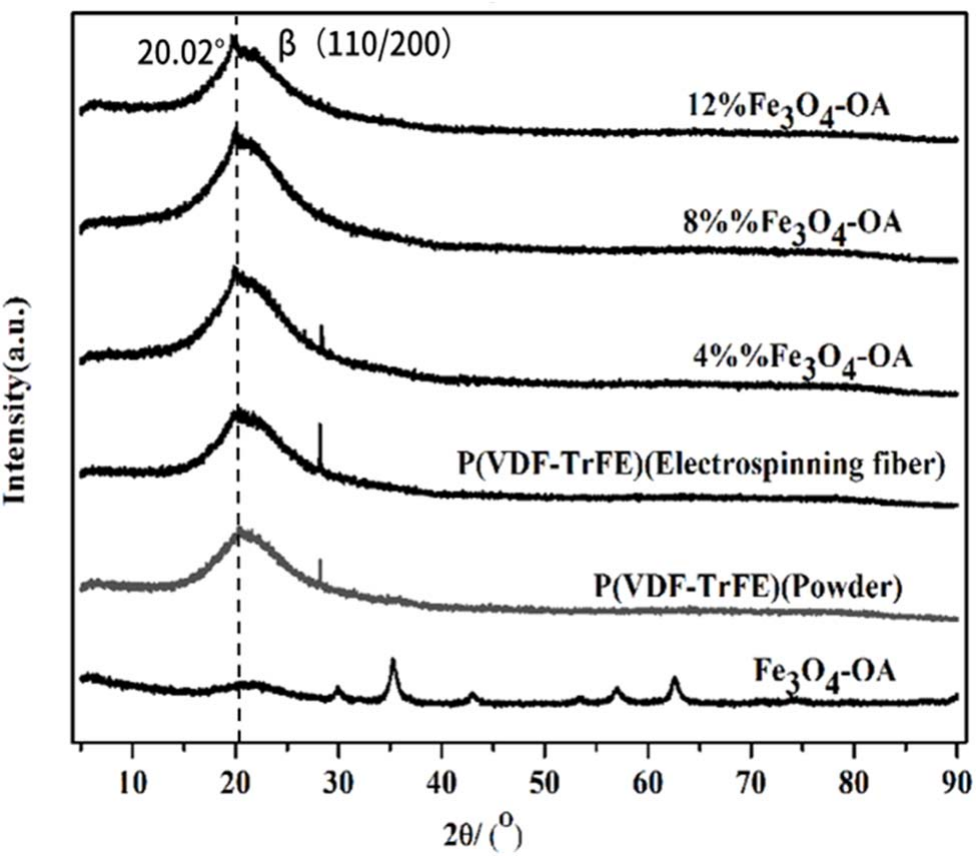
XRD patterns of the P(VDF-TrFE)/Fe_3_O_4_-OA composite electrospun membranes.

XRD was employed to characterize the crystal structures and properties of the P(VDF-TrFE)/Fe_3_O_4_-OA composite membranes with varying Fe_3_O_4_-OA contents. The XRD patterns of these composite membranes are presented in Fig. 3. Notably, it could be observed that the main diffraction peaks of the composite membranes corresponded well to those of pure P(VDF-TrFE), indicating that the incorporated Fe_3_O_4_-OA NPs did not interfere with the crystallization process of P(VDF-TrFE) or destroy its inherent crystal structure. A characteristic diffraction peak of the β-phase of P(VDF-TrFE) appeared at 2*θ* = 20.02°, a key piezoelectric phase in P(VDF-TrFE)-based materials. With an increase in the Fe_3_O_4_-OA content, the intensity of the β-phase diffraction peak of the composite membranes exhibited a trend of first increasing and then decreasing. Specifically, the diffraction peak intensity of the β-phase reached its maximum when the Fe_3_O_4_-OA content was 8 wt%. This result suggested that doping an appropriate amount of Fe_3_O_4_-OA NPs was conducive to promoting the formation and growth of the β-phase in P(VDF-TrFE). In contrast, the excessive compounding of Fe_3_O_4_-OA NPs may lead to NP aggregation in the polymer matrix, in turn inhibiting the formation of the β-phase and reducing the intensity of its diffraction peak.

### 3.3 Fourier transform infrared (FTIR) analysis

**Fig. 4:**
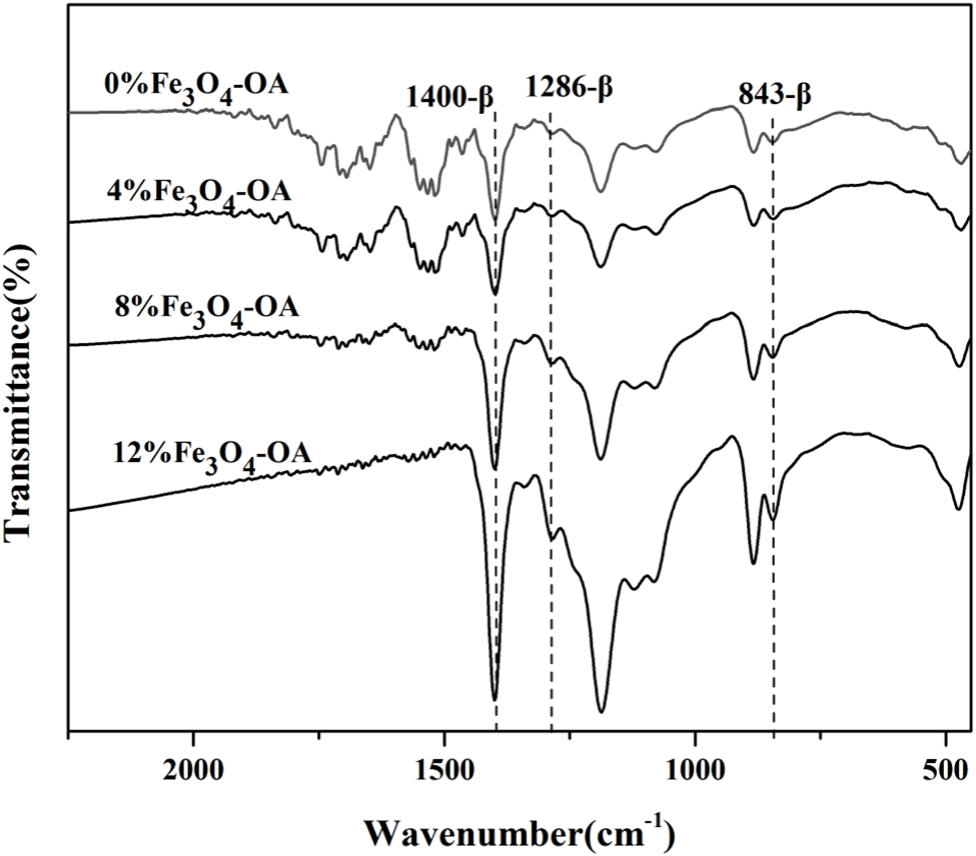
FTIR spectra of the P(VDF-TrFE)/Fe_3_O_4_-OA composite electrospun membranes.

FTIR spectroscopy was performed to characterise the chemical structure and crystalline phase composition of the samples; the spectra of the four samples are shown in Fig. 4. The absorption band at 843 cm^−1^ was assigned to the planar wagging vibration of the –CH_2_ groups and the symmetric stretching vibration of the –CF_2_ groups in P(VDF-TrFE). The peak at 1286 cm^−1^ corresponded to the asymmetric stretching vibration of the –CF_2_ groups, whereas the band at 1400 cm^−1^ was a composite peak originating from the out-of-plane wagging vibration of the –CH_2_ groups and the asymmetric stretching vibration of the C–C bonds. Notably, the strong absorption peak at 843 cm^−1^, a typical characteristic peak of the piezoelectric β-phase, was accompanied by a weak shoulder peak that was also attributable to the β-phase. The absorption peak at 1286 cm^−1^ was an additional signature of the polar ferroelectric β-phase, and the band at 1400 cm^−1^ served as another β-phase characteristic peak, whose intensity was correlated with the polar axis orientation in the polar crystalline phase. As clearly observed in the spectra, all composite membrane samples exhibited three distinct and well-defined absorption peaks corresponding to the β crystalline phase, an advantageous structural feature for excellent piezoelectric performance. In contrast, the characteristic absorption peaks of the non-polar α-phase at 766 cm^−1^ and 976 cm^−1^ were negligible in all samples. These results confirmed that the crystalline structure of the prepared P(VDF-TrFE)/Fe_3_O_4_-OA composite membranes was predominantly composed of the β-phase, laying a solid structural foundation for enhancing the piezoelectric properties of the composite materials.

### 3.4 Thermogravimetric analysis (TGA)

**Fig. 5:**
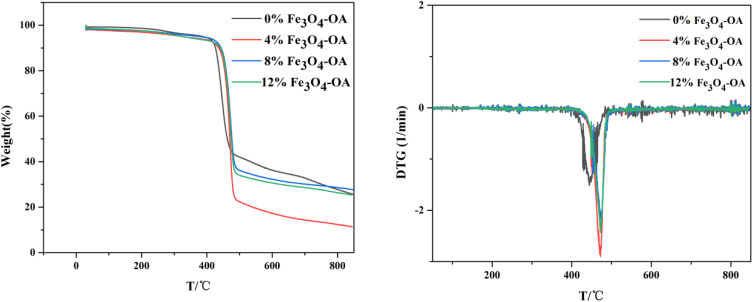
TG and DTG curves of the P(VDF-TrFE)/Fe_3_O_4_-OA composite electrospun membranes.

TGA is a widely used and reliable method for evaluating the thermal stability, heat resistance and thermal degradation behaviour of materials. Fig. 5 presents the TGA curves of the P(VDF-TrFE)/Fe_3_O_4_-OA composite membranes with four different Fe_3_O_4_-OA mass contents. Notably, the TGA curves of all samples exhibited only a single distinct weight-loss step, indicating a single-stage thermal degradation process. The main thermal degradation of all four composite membrane samples occurred in the temperature range from 400 °C to 550 °C, which was consistent with the typical thermal decomposition behaviour of P(VDF-TrFE)-based polymers. In addition, the derivative thermogravimetric (DTG) curves, which reflected the thermal degradation rate of the materials, were obtained by taking the first derivative of the TGA curves, and these DTG curves are also presented in Fig. 5. The peak temperature on each DTG curve corresponded to the temperature at which the polymer exhibited the maximum rate of thermal degradation. Based on these curves, a slight mass loss occurred during the initial heating stage (below 200 °C), which was presumably due to the evaporation of adsorbed water molecules and residual solvent in the electrospun composite membranes. During the main thermal decomposition stage of the samples, a comparison of the DTG curves of the composite membranes revealed that the initial and final thermal decomposition temperatures of the P(VDF-TrFE) matrix changed slightly before and after incorporating Fe_3_O_4_-OA NPs. However, the composite TGA temperature corresponding to the maximum decomposition rate is widely used and reliable for evaluating the thermal stability, heat resistance and thermal degradation behavior of materials. Fig. 5 presents the TGA curves of the P(VDF-TrFE)/Fe_3_O_4_-OA composite membranes with four different Fe_3_O_4_-OA mass contents. Notably, the TGA curves of all samples exhibited a single distinct weight-loss step, indicating a single-stage thermal degradation process. The main thermal degradation of all four composite membrane samples occurred in the temperature range from 400 °C to 550 °C, consistent with the typical thermal decomposition behavior of the P(VDF-TrFE)-based polymers.

Furthermore, each DTG curve exhibited only one prominent peak, indicating that all composite membranes underwent thermal decomposition through the same reaction mechanism and initiated at the same stage, suggesting that the incorporation of Fe_3_O_4_-OA NPs did not alter the intrinsic thermal degradation mechanism of the P(VDF-TrFE) polymer matrix. The temperature of the membranes increased significantly after doping with Fe_3_O_4_-OA, rising from 444 °C (pure P(VDF-TrFE)) to 472 °C. This enhancement in thermal decomposition stability can be attributed to the fact that Fe_3_O_4_-OA NPs act as physical crosslinking points in the composite material system: they restrict the thermal motion of the P(VDF-TrFE) molecular chains through physical crosslinking interactions, thereby increasing the energy required for the breakage of molecular chains.^31^

### 3.5 Differential scanning calorimetry (DSC)

**Fig. 6:**
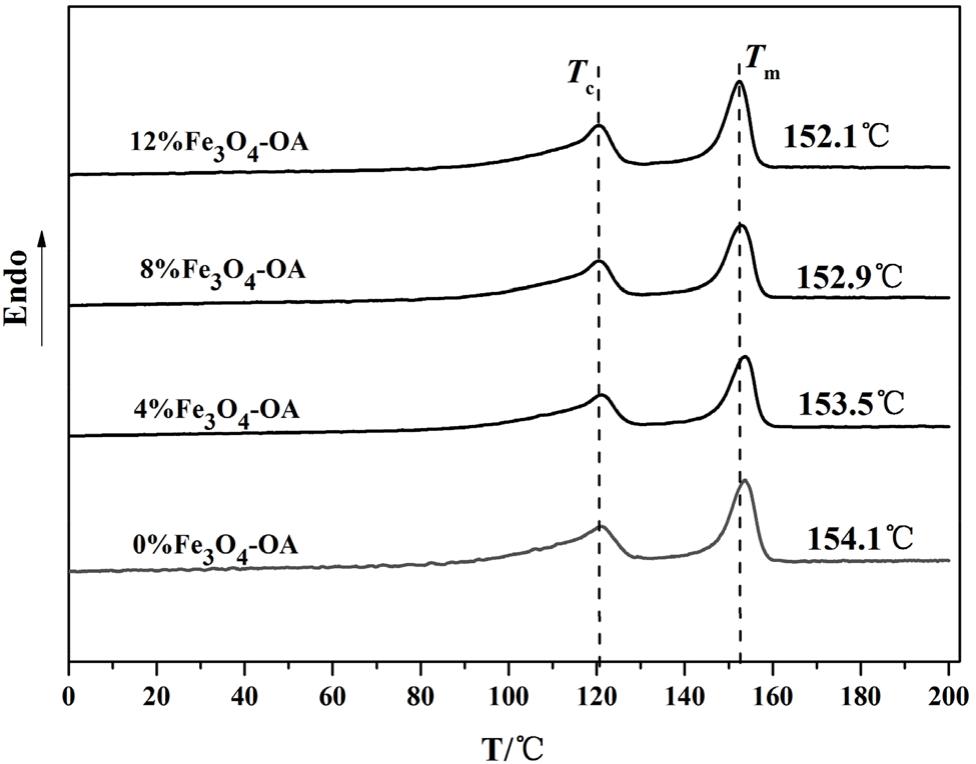
DSC curves of the P(VDF-TrFE)/Fe_3_O_4_-OA composite electrospun membranes.

By comparing the DSC curves of the P(VDF-TrFE)/Fe_3_O_4_-OA composite membranes with different Fe_3_O_4_-OA NPs contents (Fig. 6), it was observed that each curve exhibited two distinct characteristic peaks during the heating process. The first characteristic peak, corresponding to the Curie temperature (*T*_c_), was associated with the phase transition of the sample from the ferroelectric phase to the paraelectric phase. The second characteristic peak corresponded to the crystallisation melting temperature (*T*_m_) of the composite membranes. Notably, *T*_m_ of the composite membranes decreased gradually with an increase in the Fe_3_O_4_-OA NPs content, whereas *T*_c_ remained relatively unchanged. The decrease in *T*_m_ was attributable to the incorporation of Fe_3_O_4_-OA NPs, which disrupted the original intermolecular forces within the P(VDF-TrFE) matrix and increased the internal energy of the composite system, thereby reducing the energy required to melt P(VDF-TrFE) crystals (Table 1).

**Table 1:** Enthalpy of melt for different concentrations of Fe_3_O_4_-OA in P(VDF-TrFE)

Sample	0%	4%	8%	12%
Area (J g^−1^)	38.2	35.8	35.7	34.4
*X*_c_	84.9	82.9	86.2	86.9

The melting enthalpy of 100% crystalline P(VDF-TrFE) was 45 J g^−1^. The crystallinity of the P(VDF-TrFE)/Fe_3_O_4_-OA composite membranes was calculated using the following formula:*X*_c_ (%) = Δ*H*_m_/(1 − *ω*)Δ*H*_0_ × 100%,where Δ*H*_m_: enthalpy of melting of the sample, Δ*H*_0_: enthalpy of melting of 100% crystalline P(VDF-TrFE), and *ω*: the content of Fe_3_O_4_-OA in the composite fiber membrane.

The crystallinity of each P(VDF-TrFE)/Fe_3_O_4_-OA composite membrane sample was calculated using the corresponding formula. The results were 84.9% (0% Fe_3_O_4_-OA), 82.9% (4% Fe_3_O_4_-OA), 86.2% (8% Fe_3_O_4_-OA) and 86.9% (12% Fe_3_O_4_-OA). Notably, the crystallinity of the composite membranes first decreased after the initial doping of Fe_3_O_4_-OA NPs. This phenomenon was attributable to the presence of a certain amount of Fe_3_O_4_-OA NPs and the interfacial interactions between the NPs and the P(VDF-TrFE) polymer matrix, which exerted a certain inhibitory effect on the growth and development of the P(VDF-TrFE) crystals. However, with further increases in the Fe_3_O_4_-OA NPs content, the crystallinity of the composite membranes gradually increased. This occurred because the introduction of a large number of Fe_3_O_4_-OA NPs provided heterogeneous nucleation sites for the P(VDF-TrFE) molecular chains, effectively promoting the crystallization process of the polymer matrix. In summary, the crystallinity of the P(VDF-TrFE)/Fe_3_O_4_-OA composite membranes exhibited a non-monotonic variation trend of “first decreasing and then increasing” with an increase in the Fe_3_O_4_-OA NPs content.

### 3.6 Piezoelectric performance

**Fig. 7:**
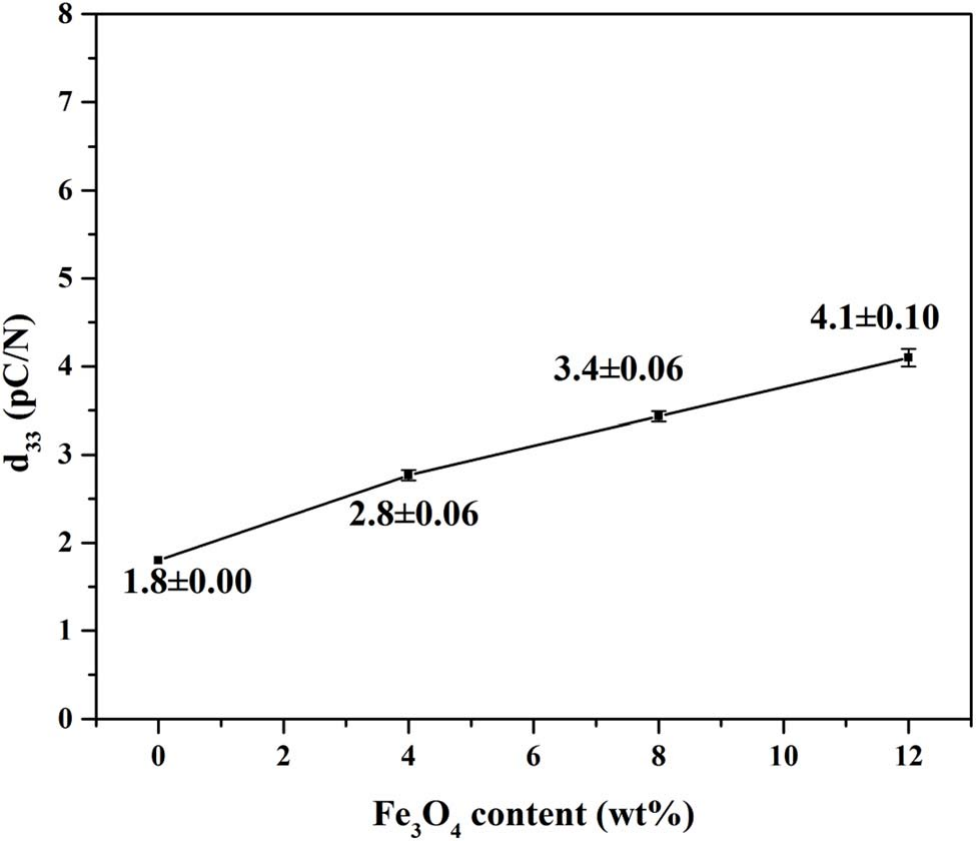
*d*_33_ Plot of the P(VDF-TrFE)/Fe_3_O_4_-OA composite electrospun membranes.

P(VDF-TrFE) is a polymeric material with excellent piezoelectric properties, and a large piezoelectric coefficient corresponds to high energy conversion efficiency. The piezoelectric coefficient, *d*_33_, is a key parameter for characterising the piezoelectric performance of piezoelectric materials. In the original state without external force application, no potential difference exists between the two electrodes of P(VDF-TrFE). When subjected to an external force, a dipole moment is generated along the strain direction, accompanied by the formation of polarised charges, inducing the accumulation of opposite charges on the electrode surface and thereby resulting in a macroscopic piezoelectric potential. As shown in Fig. 7, the P(VDF-TrFE)/12% Fe_3_O_4_-OA composite exhibited the maximum *d*_33_ value, and the piezoelectric coefficient presented an increasing trend with the increase in Fe_3_O_4_-OA content within the test range. This enhanced piezoelectric performance was attributable to the formation of numerous two-phase interface regions between Fe_3_O_4_-OA NPs and the P(VDF-TrFE) matrix. Upon introducing an appropriate amount of Fe_3_O_4_-OA, these interface regions could effectively trap more free charges generated by the deformation of the polymer matrix, and the increased charge density at the two electrodes further amplified the macroscopic piezoelectric potential output. In addition, the good dispersion of Fe_3_O_4_-OA in the polymer matrix also reduced the occurrence of charge recombination at the interface, which was conducive to the stable accumulation of polarised charges and the improvement of piezoelectric response.

### 3.7 Magnetic analysis

Hysteresis loop measurement is a key method for characterising the magnetic properties of ferromagnetic materials, and the vibrating sample magnetometer (VSM) is a commonly used instrument for testing the hysteresis loop of materials. Three core parameters can be derived from a hysteresis loop: saturation magnetisation (*M*_s_), remanent magnetisation (*M*_r_) and coercivity (*H*_c_). Saturation magnetisation is the maximum and stable magnetisation intensity achieved by magnetic materials, whose magnetisation continuously increases with an increase in external magnetic field during the magnetisation process and eventually plateaus. When the applied magnetic field is removed (*i.e.* the magnetic field strength is reduced to zero), the nonzero magnetisation intensity retained by the magnetised material is defined as remanent magnetisation. The reverse magnetic field required to reduce the remanent magnetisation to zero is termed the coercive field, or coercivity for short.

**Fig. 8:**
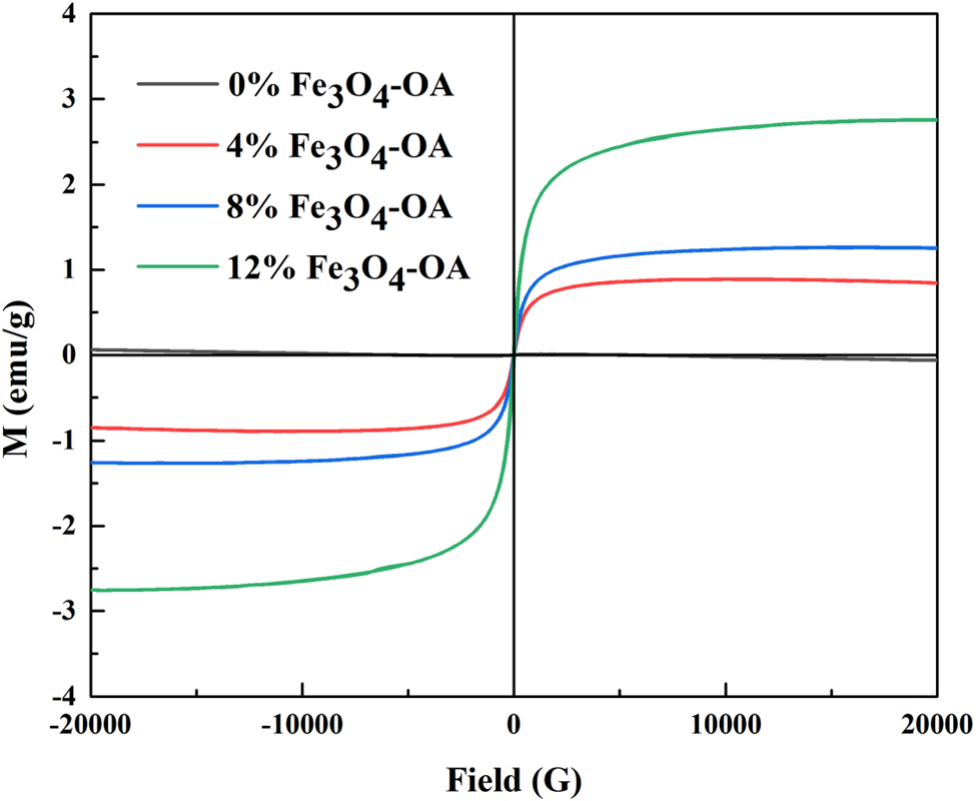
VSM curves of the P(VDF-TrFE)/Fe_3_O_4_-OA composite electrospun membranes.

As shown in Fig. 8, the saturation magnetization of the composite membranes increased from 0.87 emu per g to 1.25 emu per g and 2.75 emu per g with an increase in the mass concentration of Fe_3_O_4_-OA NPs from 4%, 8% and 12%, respectively. Within a certain range, the increase in Fe_3_O_4_-OA NPs content introduced additional magnetic particles that contributed magnetic moments, thereby leading to the gradual elevation of saturation magnetisation in the composite membranes. Meanwhile, all composite membranes exhibited relatively low coercivity, indicating that the Fe_3_O_4_-OA NPs were small single-domain particles with weak magnetic anisotropy. The remanent magnetisation of the composite membranes containing 4%, 8% and 12% Fe_3_O_4_-OA NPs was measured to be 0.030 emu per g, 0.039 emu per g and 0.083 emu per g, respectively. This trend reflects the weak magnetic interaction between Fe_3_O_4_-OA NPs in the matrix, confirming a positive correlation between the remanent magnetisation of the composite membranes and the mass concentration of Fe_3_O_4_-OA NPs.

### 3.8 Effect of Fe_3_O_4_-OA content on BMSC proliferation

BMSCs cultured on the composite membranes were maintained under static magnetic field conditions during all *in vitro* experiments. The cytocompatibility of the P(VDF-TrFE)/Fe_3_O_4_-OA composite membranes was evaluated by CCK-8 assay and live/dead staining, with cells cultured on tissue culture plates (TCP) serving as a control group.

**Fig. 9:**
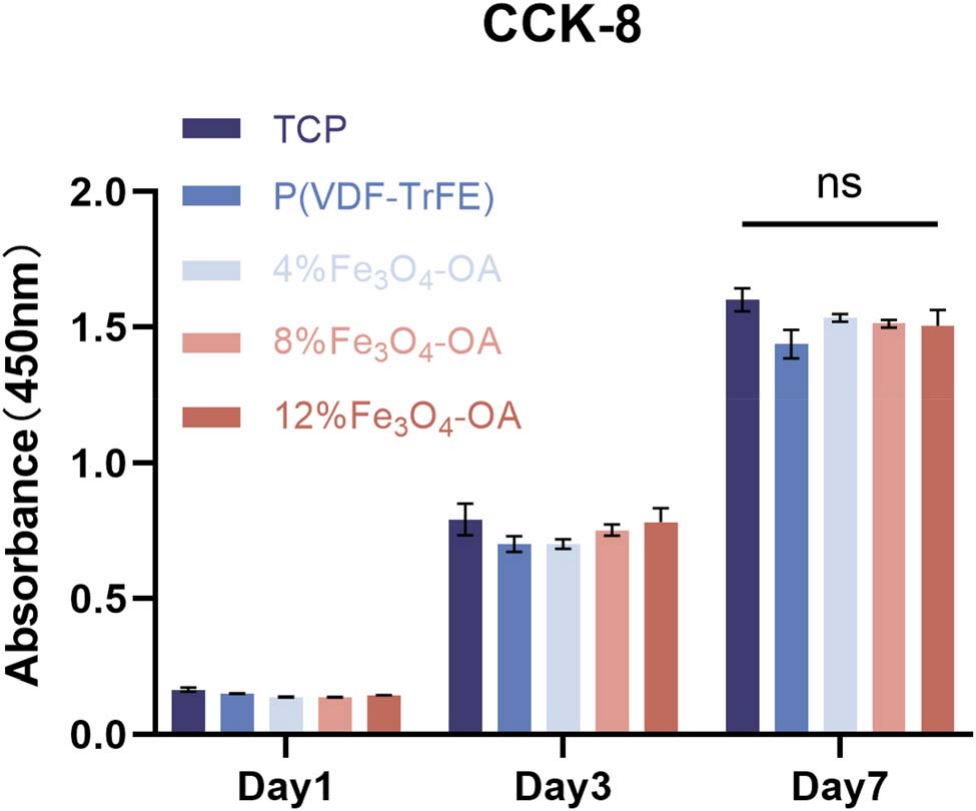
Proliferation of the BMSCs cultured on TCP or the P(VDF-TrFE)/Fe_3_O_4_-OA membranes with different Fe_3_O_4_-OA contents. Data are presented as mean ± SD (*n* = 3). Statistical analysis was performed using two-way ANOVA; ns indicates no significant difference.

The CCK-8 results showed that BMSCs maintained good proliferation on all membranes over 1, 3, and 7 days (Fig. 9). All Fe_3_O_4_-OA-containing groups exhibited comparable proliferative activity to the 0% group. Overall, cell viability in all groups remained above 90%, indicating no obvious cytotoxicity.

**Fig. 10:**
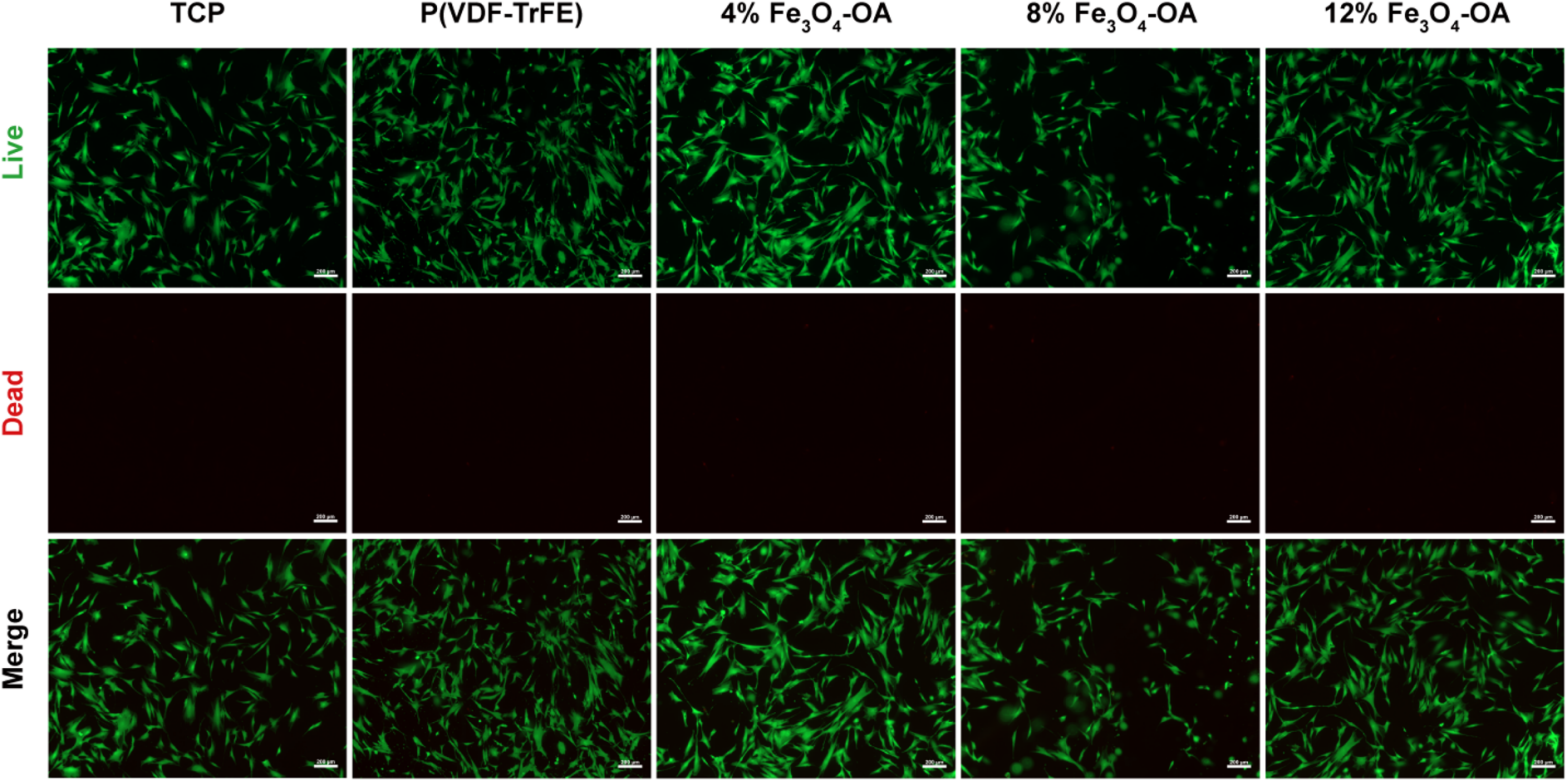
Live/dead staining of the BMSCs cultured on TCP or the composite membranes with different Fe_3_O_4_-OA contents.

Live/dead staining further confirmed high cell viability across all groups, including the TCP control, with predominant green fluorescence and minimal red fluorescence (Fig. 10). No evident differences in cell density or morphology were observed with increasing Fe_3_O_4_-OA content. These results indicated that the composite membranes exhibited good cytocompatibility and supported BMSC survival.

### 3.9 Characterisation of cell spreading on the membrane surfaces

**Fig. 11:**
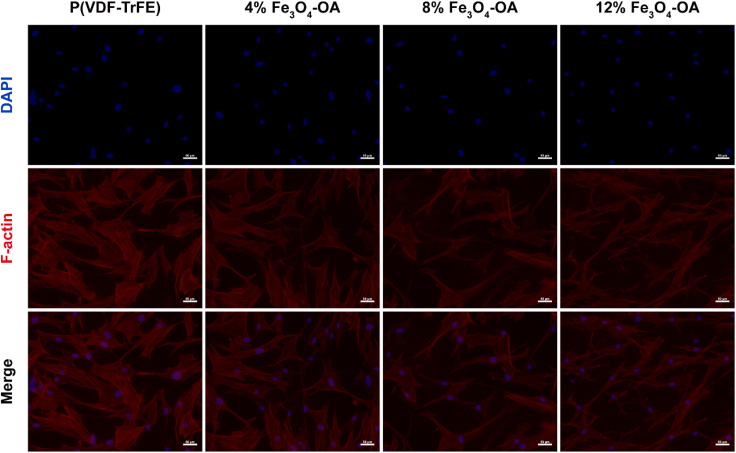
Characterization of the cell spreading of the BMSCs cultured on the composite membranes with different Fe_3_O_4_-OA contents.

To investigate the effects of Fe_3_O_4_-OA content on BMSC adhesion and osteogenesis-related morphological changes, F-actin cytoskeletal staining was conducted after 3 days of cell culture on the composite membranes (Fig. 11). BMSCs in all experimental groups exhibited favourable adhesion and extensive spreading on the membrane surfaces, indicating good cell–matrix interaction across all formulations. Notably, cells cultured on the 8% and 12% Fe_3_O_4_-OAmembranes displayed more prominent pseudopodial extension and well-developed actin stress fibers compared with the other groups. With an increase in the Fe_3_O_4_-OA content, the cellular morphology gradually transitioned from a typical spindle shape to a more flattened, spread-out morphology with multiple cellular protrusions. This cytoskeletal reorganisation is indicative of the enhanced cell substrate interactions and improved mechanosensing capacity of BMSCs, which are critical cellular events closely associated with the subsequent activation of osteogenic signalling pathways.

### 3.10 ALP activity indicates early osteogenic differentiation

**Fig. 12:**
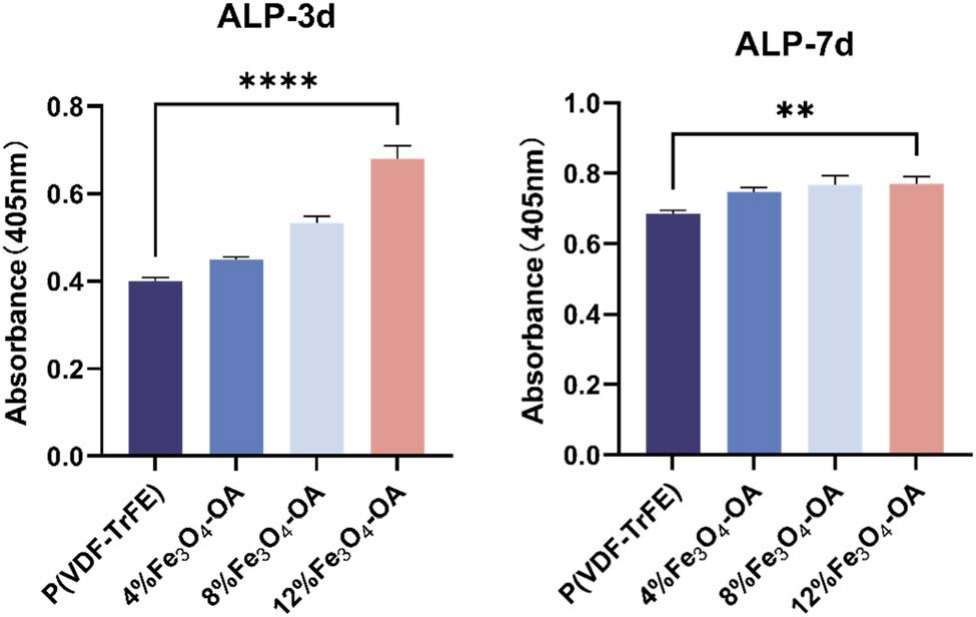
ALP activity of the BMSCs cultured on the composite membranes with different Fe_3_O_4_-OA contents.

To further evaluate the osteogenic differentiation potential of the composite membranes, alkaline phosphatase (ALP) activity, an early marker of osteogenesis, was measured in BMSCs cultured on the membranes at 3 and 7 days (Fig. 12). Compared with the 0% Fe_3_O_4_-OA group, all Fe_3_O_4_-OA-containing membranes exhibited increased ALP activity, showing a concentration-dependent trend. The 12% Fe_3_O_4_-OA group displayed the highest ALP activity at both time points, indicating enhanced early osteogenic responses with increasing Fe_3_O_4_-OA content.

These results were consistent with the F-actin staining findings, suggesting that the improved cytoskeletal organization and cell adhesion may contribute to the enhanced osteogenic responses. The concentration-dependent increase in ALP activity indicated that Fe_3_O_4_-OA incorporation was associated with the promotion of early-stage osteogenic differentiation in BMSCs.

### 3.11 RUNX2 immunofluorescence reveals the activation of osteogenic transcription

**Fig. 13:**
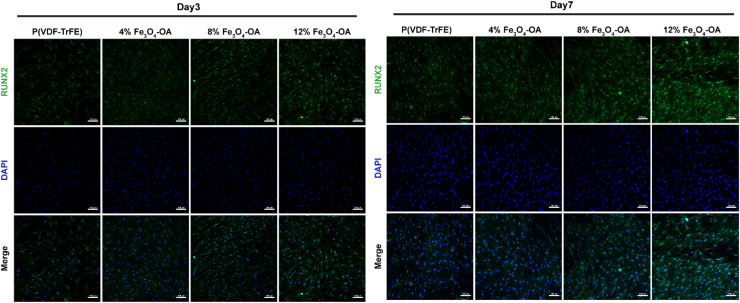
Immunofluorescence staining of RUNX2 in the BMSCs cultured on the composite membranes. RUNX2 expression and nuclear localisation increased with the Fe_3_O_4_-OA content on days 3 and 7, indicating enhanced osteogenic transcriptional activation.

To elucidate the transcriptional basis of this osteogenic activation, the immunofluorescence staining of runt-related transcription factor 2 (RUNX2), a master transcription factor governing osteogenic lineage commitment, was performed to investigate the transcriptional activation of osteogenic differentiation (Fig. 13). At day 3, markedly enhanced RUNX2 expression and distinct nuclear localisation were already observed in the 8% and 12% Fe_3_O_4_-OA groups, indicating the early initiation of osteogenic transcriptional regulation by Fe_3_O_4_-OA incorporation. By day 7, RUNX2 expression was significantly upregulated in all Fe_3_O_4_-OA-containing groups relative to the 0% control group, with the 12% Fe_3_O_4_-OA group exhibiting the strongest fluorescent signal. These molecular findings were consistent with the aforementioned morphological and functional results, suggesting coordinated changes during early osteogenic differentiation. Fe_3_O_4_-OA incorporation enhanced BMSC adhesion and cytoskeletal remodeling, which may contribute to the upregulation of RUNX2 and its downstream transcriptional program, and was associated with enhanced osteogenic differentiation. Collectively, these results suggested that the Fe_3_O_4_-OA-modified P(VDF-TrFE) composite membranes supported the early stage of osteogenic differentiation in BMSCs.

### 3.12 Osteogenic protein expression in BMSCs cultured on the composite membranes

**Fig. 14:**
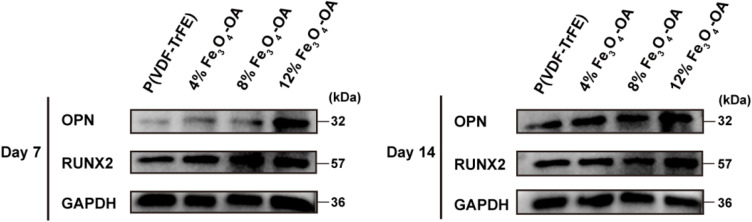
Western blot analysis revealing the concentration-dependent upregulation of canonical osteogenic markers (RUNX2 and OPN) on the composite membranes.

To further evaluate osteogenic differentiation at the protein level, Western blot analysis was performed to quantify the expression of RUNX2 and osteopontin (OPN), key markers of early and late-stage osteogenesis, respectively, at 7 and 14 days (Fig. 14). At day 7, both RUNX2 and OPN expression levels were increased in all Fe_3_O_4_-OA-containing groups compared with the 0% group, showing a concentration-dependent trend. By day 14, the expression of these proteins was further elevated, with the highest levels observed in the 12% Fe_3_O_4_-OA group.

These results were consistent with the immunofluorescence, ALP activity, and F-actin staining findings, suggesting that Fe_3_O_4_-OA incorporation was associated with sustained osteogenic responses in BMSCs. The increased expression of RUNX2 and OPN indicated enhanced osteogenic differentiation from early to later stages.

### 3.13 Alizarin Red S (ARS) staining confirms late-stage osteogenic mineralization

**Fig. 15:**
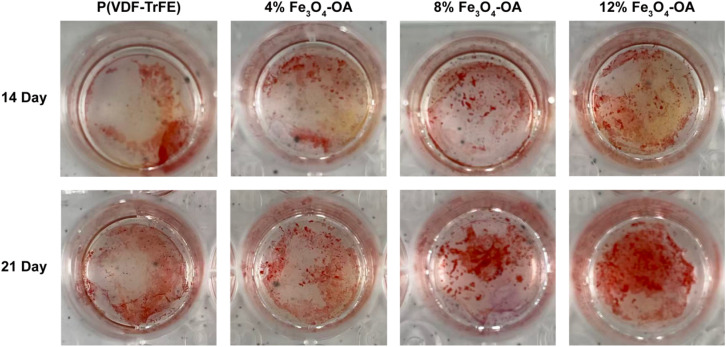
ARS staining of the mineralized matrix formed by the BMSCs cultured on the P(VDF-TrFE)/Fe_3_O_4_-OA composite membranes.

To evaluate late-stage osteogenic differentiation and ECM mineralization, ARS staining was performed to visualize calcium deposition (Fig. 15). Compared with the 0% Fe_3_O_4_-OA group, BMSCs cultured on the Fe_3_O_4_-OA-containing membranes exhibited increased calcium deposition, showing a concentration-dependent trend. The 12% Fe_3_O_4_-OA group displayed the highest level of mineralization, with dense and uniformly distributed calcium nodules, whereas only limited staining was observed in the 0% group.

These results were consistent with the Western blot data of osteogenic proteins, suggesting that Fe_3_O_4_-OA incorporation was associated with enhanced extracellular matrix mineralization. The increased calcium deposition indicated that the composite membranes supported the progression of osteogenic differentiation toward later stages in a concentration-dependent manner. While the above findings provided useful insights, it should be noted that the present study was conducted under the static magnetic field conditions used here, and the conclusions primarily reflected the relative differences among composite membranes with varying Fe_3_O_4_-OA contents.

## 4. Conclusions

In this study, magnetoactive electrospun P(VDF-TrFE) membranes loaded with different ratios of Fe_3_O_4_-OA NPs were fabricated, and their interaction with BMSCs and normal human cells was comprehensively studied. P(VDF-TrFE)/Fe_3_O_4_-OA scaffolds showed defect-free morphology, with an average fiber diameter of ∼0.23 µm, and a homogeneous distribution of the magnetic filler within the polymer owing to a stabilising effect of the OA molecules. As evidenced by XRD and FTIR spectroscopy (phase composition analysis), the P(VDF-TrFE)/Fe_3_O_4_-OA membranes predominantly contained an electroactive β-phase. The incorporation of 12 wt% of Fe_3_O_4_-OA NPs into the P(VDF-TrFE) membranes allowed us to achieve, to the best of our knowledge, the highest *M*_s_ of 0.083 emu per g and a nonzero *H*_c_, which was characteristic of ferromagnetic materials. Cell-based experiments further confirmed that the adhesion of BMSCs to the P(VDF-TrFE)/Fe_3_O_4_-OA composite membranes effectively supported osteogenic responses throughout the early and late stages of differentiation. Specifically, BMSCs cultured on the composite membranes exhibited enhanced spreading and well-organized cytoskeletal structures, indicating improved cell–substrate interactions, which were associated with osteogenic differentiation. Functional evaluations showed increased ALP activity, along with the elevated expression of osteogenic markers (RUNX2 and OPN), at different time points, suggesting enhanced osteogenic responses in BMSCs. In addition, the formation of a mineralized ECM, as evidenced by ARS staining, further indicated improved late-stage osteogenic differentiation and mineralization. Taken together, these results suggest that the magnetoactive P(VDF-TrFE)/Fe_3_O_4_-OA composite membranes provide a favorable microenvironment for BMSC osteogenic differentiation, indicating their potential application in bone tissue engineering.

## Author contributions

Na Qiang: writing – original draft; Lihong Huang: formal analysis; Wenlong Zhang: visualization; Rouping Zheng: experiment and investigation; Yan Yu: investigation; Jiao Zou: software; Qingyun Tang: dData curation; Yubo Zou: validation; Jinyu Yang: methodology; Guocong Liu: project administration; Shuai Qiu: writing – review and editing; and Shuo Tang: supervision.

## Conflicts of interest

The authors declare that there are no conflicts of interest.

## Supplementary Material

RA-016-D6RA01554A-s001

## Data Availability

Data will be made available upon request. Supplementary information is available. See DOI: https://doi.org/10.1039/d6ra01554a.
