# Supplementary figures and images for: Fabrication and osteogenic differentiation performance of the electrospun magnetic P(VDF-TrFE)/Fe3O4 composite fibrous membranes
Source: RSC Adv. 2026 May 27;16(31):28604–18. doi: 10.1039/d6ra01554a (PMC13216860; doi:10.1039/d6ra01554a)

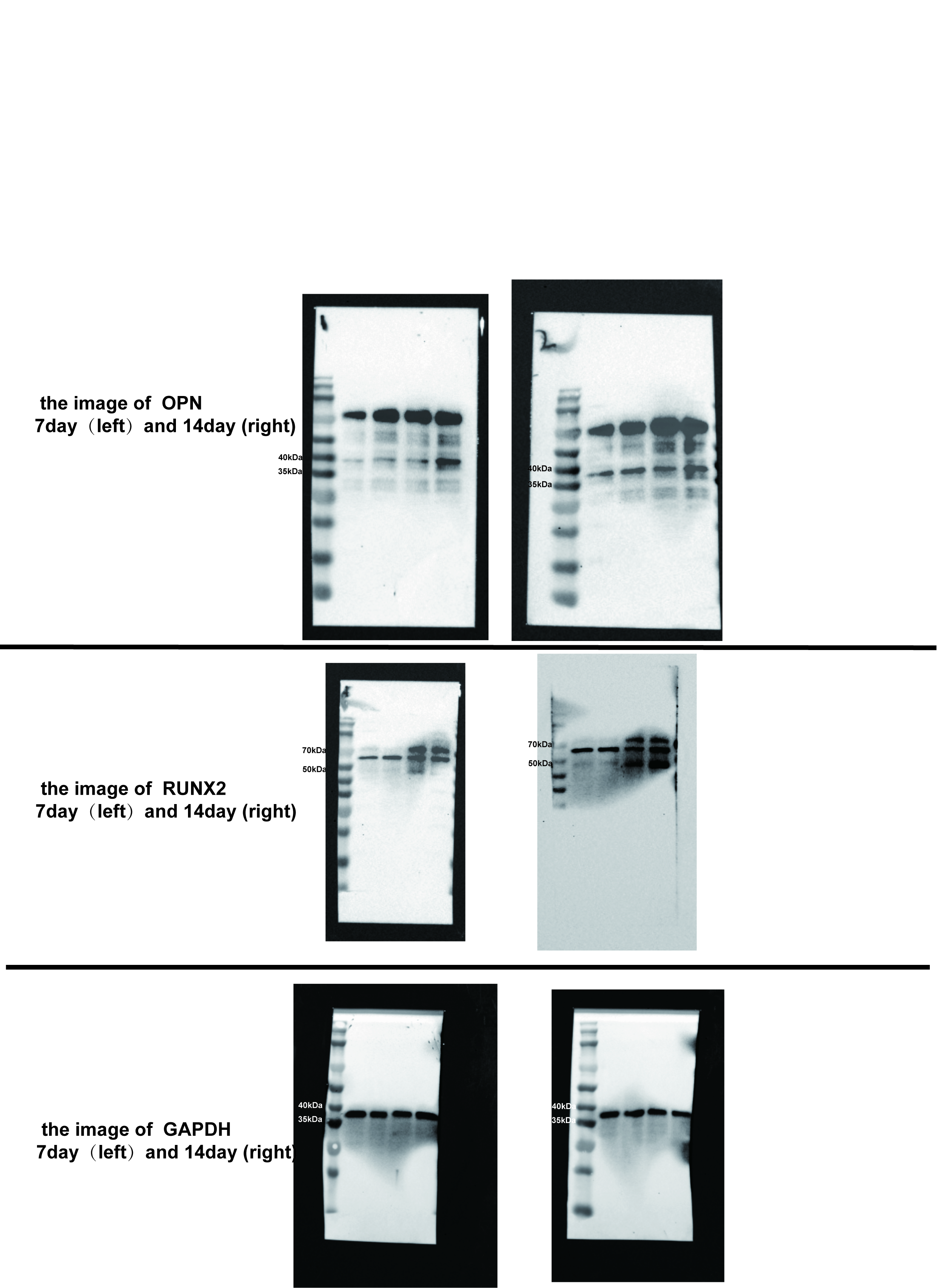

Supplement: RA-016-D6RA01554A-s001 [file RA-016-D6RA01554A-s001.zip › supplementary information.tif]
